# SREB2/GPR85, a schizophrenia risk factor, negatively regulates hippocampal adult neurogenesis and neurogenesis-dependent learning and memory

**DOI:** 10.1111/j.1460-9568.2012.08180.x

**Published:** 2012-09

**Authors:** Qian Chen, Jeffrey H Kogan, Adam K Gross, Yuan Zhou, Noah M Walton, Rick Shin, Carrie L Heusner, Shinichi Miyake, Katsunori Tajinda, Kouichi Tamura, Mitsuyuki Matsumoto

**Affiliations:** 1 CNS, Astellas Research Institute of America LLCSkokie, IL 60077, USA; 2 Master of Biotechnology Program, Northwestern UniversityEvanston, IL, USA

**Keywords:** bromodeoxyuridine, cognition, dentate gyrus, mutant mouse, psychiatric disease

## Abstract

SREB2/GPR85, a member of the super-conserved receptor expressed in brain (SREB) family, is the most conserved G-protein-coupled receptor in vertebrate evolution. Previous human and mouse genetic studies have indicated a possible link between SREB2 and schizophrenia. SREB2 is robustly expressed in the hippocampal formation, especially in the dentate gyrus, a structure with an established involvement in psychiatric disorders and cognition. However, the function of SREB2 in the hippocampus remains elusive. Here we show that SREB2 regulates hippocampal adult neurogenesis, which impacts on cognitive function. Bromodeoxyuridine incorporation and immunohistochemistry were conducted in SREB2 transgenic (Tg, over-expression) and knockout (KO, null-mutant) mice to quantitatively assay adult neurogenesis and newborn neuron dendritic morphology. Cognitive responses associated with adult neurogenesis alteration were evaluated in SREB2 mutant mice. In SREB2 Tg mice, both new cell proliferation and new neuron survival were decreased in the dentate gyrus, whereas an enhancement of new neuron survival occurred in SREB2 KO mouse dentate gyrus. Doublecortin staining revealed dendritic morphology deficits of newly generated neurons in SREB2 Tg mice. In a spatial pattern separation task, SREB2 Tg mice displayed a decreased ability to discriminate spatial relationships, whereas SREB2 KO mice had enhanced abilities in this task. Additionally, SREB2 Tg and KO mice had reciprocal phenotypes in a Y-maze working memory task. Our results indicate that SREB2 is a negative regulator of adult neurogenesis and consequential cognitive functions. Inhibition of SREB2 function may be a novel approach to enhance hippocampal adult neurogenesis and cognitive abilities to ameliorate core symptoms of psychiatric patients.

## Introduction

Super-conserved receptor expressed in brain (SREB) is the most evolutionarily conserved G-protein-coupled receptor family; the family consists of SREB1 (GPR27), SREB2 (GPR85), and SREB3 (GPR173) (Matsumoto *et al.*, 2000). SREB2 shows the highest conservation rate among all G-protein-coupled receptors encoded in the genome; humans, rats and mice share a 100% identical amino acid sequence (Hellebrand *et al.*, 2000). In addition, the mRNA expression profile of SREB family members in the central nervous system is also highly conserved among mammalian species (Matsumoto *et al.*, 2000). SREB2 is expressed in all neurons, with the hippocampal dentate gyrus showing the highest expression levels of SREB2 mRNA in both humans and rodents (Matsumoto *et al.*, 2005). Although endogenous ligands for SREB family members have not yet been identified, the extremely high levels of evolutionary conservation in both their amino acid sequence and expression profile indicate fundamental roles for the SREB family in the central nervous system.

The hippocampal dentate gyrus is one of two brain regions retaining the ability to generate new neurons throughout adulthood, which bestows a unique structural and functional plasticity in the hippocampus (Abrous *et al.*, 2005; Ehninger & Kempermann, 2008; Aimone *et al.*, 2009; Eichenbaum & Fortin, 2009). A compelling number of studies have shown that psychiatric disorders are marked by a diminished size of the hippocampal formation, which may contribute to functional deficits, such as learning and memory impairment and mood dysregulation (Geuze *et al.*, 2005; Lucassen *et al.*, 2006). Disrupted adult neurogenesis is increasingly considered as a factor in the pathogenesis of psychiatric disorders (Kempermann *et al.*, 2008; Zhao *et al.*, 2008). Some interventions, such as environment enrichment, exercise, and antidepressant treatment, increase adult neurogenesis, accompanied by beneficial effects on cognition and mood regulation (Ehninger & Kempermann, 2003; Santarelli *et al.*, 2003; Olson *et al.*, 2006; Silva *et al.*, 2008; Fabel *et al.*, 2009). Thus, targeting adult neurogenesis may provide an alternative approach to restore hippocampal plasticity and prevent or alleviate behavioral symptoms in psychiatric disorders (Dhikav & Anand, 2007; DeCarolis & Eisch, 2010).

SREB2 Tg mice have been proposed as a novel animal model of schizophrenia. These mice have a reduced brain size, sensory gating deficits, social interaction deficits and cognitive impairments. Human genetic studies have revealed that patients with schizophrenia with risk haplotypes of SREB2 showed an allele load association with reduced hippocampal volume (Matsumoto *et al.*, 2008). However, the mechanism by which SREB2 confers such a complex phenotype in the transgenic animal model and human patients has not been elucidated. In the present study, we have investigated whether SREB2 plays a role in hippocampal adult neurogenesis and neurogenesis-related cognitive behaviors using SREB2 Tg (over-expression) and KO (null-mutation) mice. Our results indicate that SREB2 negatively regulates hippocampal adult neurogenesis and cognitive function. We suggest that SREB2 antagonism may be a novel therapy for treating psychiatric disorders by elevating hippocampal adult neurogenesis.

## Materials and methods

### SREB2 Tg and SREB2 KO mice

The SREB2 Tg and KO mice were generated as described previously (Matsumoto *et al.*, 2008). In SREB2 Tg mice, SREB2 cDNA and the 3′ untranslated region (UTR) region were cloned under the calcium/calmodulin dependent protein kinase II alpha (CaMKIIα) promoter. The transgene construct was integrated into the genome through pro-nuclear microinjection. In SREB2 KO mice, the endogenous SREB2 coding sequence was entirely replaced by a neomycin selection marker gene. Both SREB2 Tg and SREB2 KO mice were backcrossed more than 10 generations to the C57BL/6 strain. Mice were housed in a barrier facility and maintained under a 12 : 12 light/dark cycle in a temperature- and humidity-controlled room. Standard laboratory diet and acidified water were available *ad libitum*. All procedures were approved by the institutional laboratory animal care and use committee at Astellas Research Institute of America. The SREB2 Tg mice were bred with SREB2 Tg ♂ × C57BL/6 ♀, and SREB2 KO mice were bred with SREB2^+/−^♂ × SREB2^+/−^♀. Only male offspring with the genotype of Tg^+^ (SREB2 Tg) and Tg^−^ [wild-type (WT)], SREB2^−/−^ (SREB2 KO) and SREB2^+/+^ (WT) were used in the experiments. The experimental mice were 4–6 months old.

### Quantitative reverse transcription-polymerase chain reaction for gene expression analysis in brain samples

The mice were anesthetized in a carbon dioxide chamber, then rapidly decapitated with surgical scissors. The brains were quickly removed from the skulls and kept in ice cold culture dish for dissection. The hippocampal regions of SREB2 mutant and WT littermate mice were dissected out and flash frozen on dry ice. Total RNA was isolated by using a mini RNAeasy kit (Qiagen). cDNA was synthesized according to the Superscript III kit (Invitrogen) by using a random hexamers primer. Quantitative polymerase chain reaction (PCR) was conducted by using SYBR® green PCR master mix in a ViiA real-time PCR machine (Applied Biosystem). A standard curve of each gene of interest was generated to quantify the amplification. All amplification data were normalized with glyceraldehyde-3-phophate dehydrogenase (GAPDH) for gene expression analysis.

### Primers for quantitative reverse transcription-polymerase chain reaction gene expression examination

**Table:** 

Gene	Primer
SREB2	5′-CCAGTCCAGTTTGTAGCAGCA-3′
	5′-AGGGCCATGAAAGGTCCAGT-3′
SREB1	5′-CTGAAAGGCATTGGTTTGTGAAGC-3′
	5′-TGCGTGTCCACTTTCAGAGTCTCC-3′
SREB3	5′-GGCTTTCCCACCTGTCTTTG-3′
	5′-CACTGGTCCTCCTCTCGGATAA-3′
Calbindin	5′-GCTCCGCGCACTCTCTCAA-3′
	5′-TGACTGCAGGTGGGATTCTG-3′
Desmoplakin 1	5′-GCTCCATTACCAAGACTTCATC-3′
	5′-TGTCGTCGTCTCCAAACATCT-3′
Tryptophan-2,3-dioxygenase	5′-GGCAGAGTTCCGGAAGCA-3′
	5′-CATGACGCTTCTCATCAAACAA-3′
Interleukin-1 receptor type 1	5′-CCTTCAACCAAAAGAAAATACACAC-3′
	5′-GGATAGCGATAAAACTGGCT-3′

### Mouse hippocampal neuronal stem cell culture

Hippocampal neuronal stem cells (NSCs) were isolated, cultured, and differentiated according to previous publications (Scheffler *et al.*, 2005; Babu *et al.*, 2011). In brief, the hippocampal regions were microdissected from the surrounding cortex and third ventricle of post-natal day 5 C57BL/6 pups. Mouse hippocampal NSCs were propagated in growth medium consisting of neurobasal medium supplement with B27, 5% fetal bovine serum (FBS), 2 mm Glutamax, 20 ng/mL epidermal growth factor (EGF), 20 ng/mL basic fibroblast growth factor (bFGF) and 0.5% v/v bovine pituitary extract. Serum, epidermal growth factor (EGF) and basic fibroblast growth factor (bFGF) were removed from the culture medium to induce differentiation. SREB gene expression was examined at 3 days after differentiation. The proliferated and differentiated NSCs were rinsed with phosphate-buffered saline (PBS) and lysed with RNA tissue lysis (RTL) buffer for total RNA preparation by using the Micro RNAeasy kit (Qiagen).

### Bromodeoxyuridine administration and brain tissue sample preparation

To determine cell proliferation, mice were injected three times with bromodeoxyuridine (BrdU) (100 mg/kg, i.p.) at 2 h intervals. At 3 days after the last BrdU injection, brain tissue was collected for an adult neurogenesis proliferation study. Animals were anesthetized and transcardially perfused with PBS followed by ice-cold 4% paraformaldehyde PBS solution. Brains were removed and fixed in 4% paraformaldehyde PBS solution overnight at 4 °C, cryoprotected in 30% sucrose in PBS, embedded using optimal cutting temperature (OCT) embedding compound and stored at −80 °C. To determine long-term cell survival and maturation, animals were killed at 30 days after the final injection of BrdU. The brain tissue preparation was carried out as described above.

### Immunohistochemistry staining

A floating staining protocol and immunofluorescent method were used in all immunohistochemistry staining. For BrdU immunohistochemistry, pre-treatment of samples was performed as described previously (Chen *et al.*, 2008). DNA was denatured by incubating the sections in 50% formamide in 2 × saline-sodium citrate (SSC) buffer at 65 °C for 2 h. The sections were rinsed twice with 2 × saline-sodium citrate (SSC) buffer and incubated in 2 N HCl at 37 °C for 30 min, followed by immunofluorescent staining. After extensive washes in PBS, sections were blocked with a PBS solution containing 0.3% Triton-X 100, 1% bovine serum albumin and 3% normal donkey serum for 1 h at room temperature (22–24 °C). Primary antibodies were applied overnight at 4 °C. For fluorescence immunodetection, sections were washed extensively and incubated with fluorochrome-conjugated species-specific antibodies.

The following antibodies and final dilutions were used. Primary antibodies: rat anti-BrdU (1 : 800; Abcam, UK), mouse anti-neuronal specific nuclear protein (NeuN) (1 : 500; Chemicon, Temecula, CA, USA), rabbit anti-doublecortin (DCX) (1 : 500; Chemicon) and goat anti-calretinin (1 : 2000; Millipore, CA, USA). Secondary antibodies: donkey anti-rat, anti-mouse, anti-rabbit and anti-goat conjugated with Cy3 and Cy2 (1 : 500; Jackson Immuno Research, West Grove, PA, USA).

### Quantification of bromodeoxyuridine-positive and bromodeoxyuridine/neuronal specific nuclear protein (NeuN)-positive cells

Systematic random sampling methods were applied to the sampling of the dentate gyrus. Coronal serial free-floating sections were cut at 30 μm thickness and collected through the entire hippocampus region. Every sixth section (180 μm interval) of the serial cutting sections was selected from each brain and processed for BrdU staining (3 days post-BrdU injection) or BrdU/NeuN double staining (30 days post-BrdU injection). BrdU-labeled cells were counted by using a BZ-9000 microscope (Keyence), which was equipped with a charge couple device (CCD) camera and an electronic XYZ axes stage. The resulting numbers were multiplied by 6 to obtain the estimated total number of BrdU-positive cells in the dentate gyrus subgranule zone. In BrdU/NeuN double-stained sections, BrdU-labeled cells were counted and categorized as immune-reactive for the neuronal marker NeuN. BrdU/NeuN-positive cells were further confirmed with a confocal microscope (Nikon) equipped with a laser emitting at 488, 543 and 630 nm. The Z-stack images of double-stained cells with 0.5 μm optical intervals were analyzed by double-channel histogram intensity analysis. All cell counting was conducted in ‘real-time’ fashion on a computer monitor.

### Doublecortin-positive cell counting and dendritic morphology analysis

Doublecortin-positive cells were imaged using a digital microscope (BZ-9000, Keyence). DCX-positive cells were counted exhaustively throughout the rostro-caudal extent of the granule cell layer, using a 40 × objective in serials of every sixth section from all animals. The resulting numbers were multiplied by 6 to obtain the estimated total number of DCX-labeled cells per brain. The differentiated new neurons were labeled by DCX and calretinin double staining. Positive, double-stained cells with vertically orientated dendrites, extending through the molecular layer, were selected for morphology analysis. For the production of images with an extended depth of field, the sequential Z-stack images were taken with 2 μm optical Z sections. DCX-positive cells with tertiary, relatively untruncated dendritic branches were traced on a computer monitor connected to a microscope. The tracing was guided by Keyence dimension quantitative measurement software. The dendritic length and number of intersections (branch points) were automatically calculated by a BZ-II analyzer (Keyence).

### Pattern separation test

The apparatus for this task consisted of an open field (96 cm L × 46 cm W; walls 11 cm H) and two distinct objects (5.5–8 cm H × 8–14 cm W) that were used as stimuli. Spatial pattern separation was measured with a variation of the metric spatial-processing task. In the sample trial, mice were placed in the center of an open field that contained two objects placed 20 cm apart. The mouse was allowed 20 min of free exploration of the objects. During a 5 min inter-trial interval the mouse was returned to its homecage and the objects were repositioned at 35 cm (low separation), 45 cm (medium separation), or 55 cm (high separation) apart. After the inter-trial interval, the mouse was returned to the open field for a 5 min test trial, during which it was able to reinvestigate the objects in their new orientation. The time that the mouse spent exploring the objects was recorded during both the sample and test trials. An investigation ratio was calculated as: (exploration during the 5 min test trial)/(exploration during the 5 min test trial + exploration during the last 5 min of the sample trial). Each mouse was tested on each of the object separations, in a counter-balanced design, during different sessions over the course of several days. A two-way (genotype × separation) repeated-measures anova with a Bonferroni’s multiple comparison test was performed on data collected during this task.

### Working memory test

The apparatus for the spontaneous delayed alternation test was a Y-maze (arms: 35 cm L × 7.3 cm W; walls: 13 cm H; central hub: 8.5 cm W; MedAssociates, St Albans, VT, USA). Each arm of the maze contained an automated guillotine door adjacent to the central hub. This test is based on the natural tendency of mice to explore a novel environment. When placed in a Y-maze, mice with intact working memory function will explore the least recently visited arm and thus tend to alternate visits among the three arms. The task began with a mouse placed randomly in one of the maze arms. After a 30 s maze acclimation period all of the maze doors were opened. The doors all shut again after the mouse entered any arm. The mouse was confined to the chosen arm for the duration of the delay period (20, 40, or 60 s). After the delay, the arms reopened and the mouse was free to explore the maze and enter any arm, after which the doors closed for the inter-trial interval of 60 s. Each subject received five trials at a pre-determined delay in a single session. Each mouse was tested in three different daily sessions to determine its working memory performance at each delay. A one-way anova with a Bonferroni’s multiple comparison test was performed on data collected during this task.

## Results

### SREB gene expression in the hippocampus of SREB2 Tg/KO mice

**Fig. 1:**
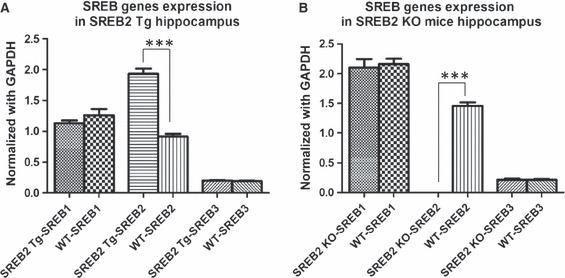
SREB family gene expression in SREB2 Tg and SREB2 KO mouse hippocampus. (A) There is a two- to threefold increase of SREB2 gene expression in the SREB2 Tg mouse (*n* = 6) hippocampus compared with WT littermates (*n* = 6) (***P < 0.0001). (B) No SREB2 transcript was detected in the SREB2 KO mouse (*n* = 6) hippocampus region compared with WT littermates (*n* = 6) (***P < 0.0001). No alteration in the expression of SREB1 and SREB3 was observed in either SREB2 Tg or SREB2 KO mice compared with their WT littermates. Data are expressed as mean values ± SEM.

The SREB family genes have a high homology of amino acid sequences and have a very similar gene expression pattern in the brain (Matsumoto *et al.*, 2000). SREB2 transgenic mice were generated by using calcium/calmodulin dependent protein kinase II alpha (CaMKIIα) promoter-driven transgene expression. The over-expression of SREB2 was restricted to neurons in the post-natal forebrain region of SREB2 Tg mice. Quantitative reverse transcription-PCR was employed to measure SREB gene expression in the hippocampus region. SREB2 Tg mice have a two- to threefold increase in SREB2 mRNA expression in the hippocampus (SREB2 Tg vs. WT, *n* = 6 of each group, two-tailed *t* = 10.68, *P* < 0.0001; Fig. 1A). Nano liquid chromatography/mass spectrometer analysis also revealed about a threefold increase of SREB2 protein expression in SREB2 Tg mouse forebrain (Yuri *et al.*, 2011). In SREB2 KO mice, the complete ablation of SREB2 expression was verified in the SREB2 KO mouse hippocampus (SREB2 KO vs. WT, *n* = 6 of each group, two-tailed *t* = 24, *P* < 0.0001; Fig. 1B). There was no apparent SREB1 or SREB3 gene expression change in either the SREB2 Tg or SREB2 KO mice.

### SREB genes are highly expressed in differentiated hippocampal neural stem cells

**Fig. 2:**
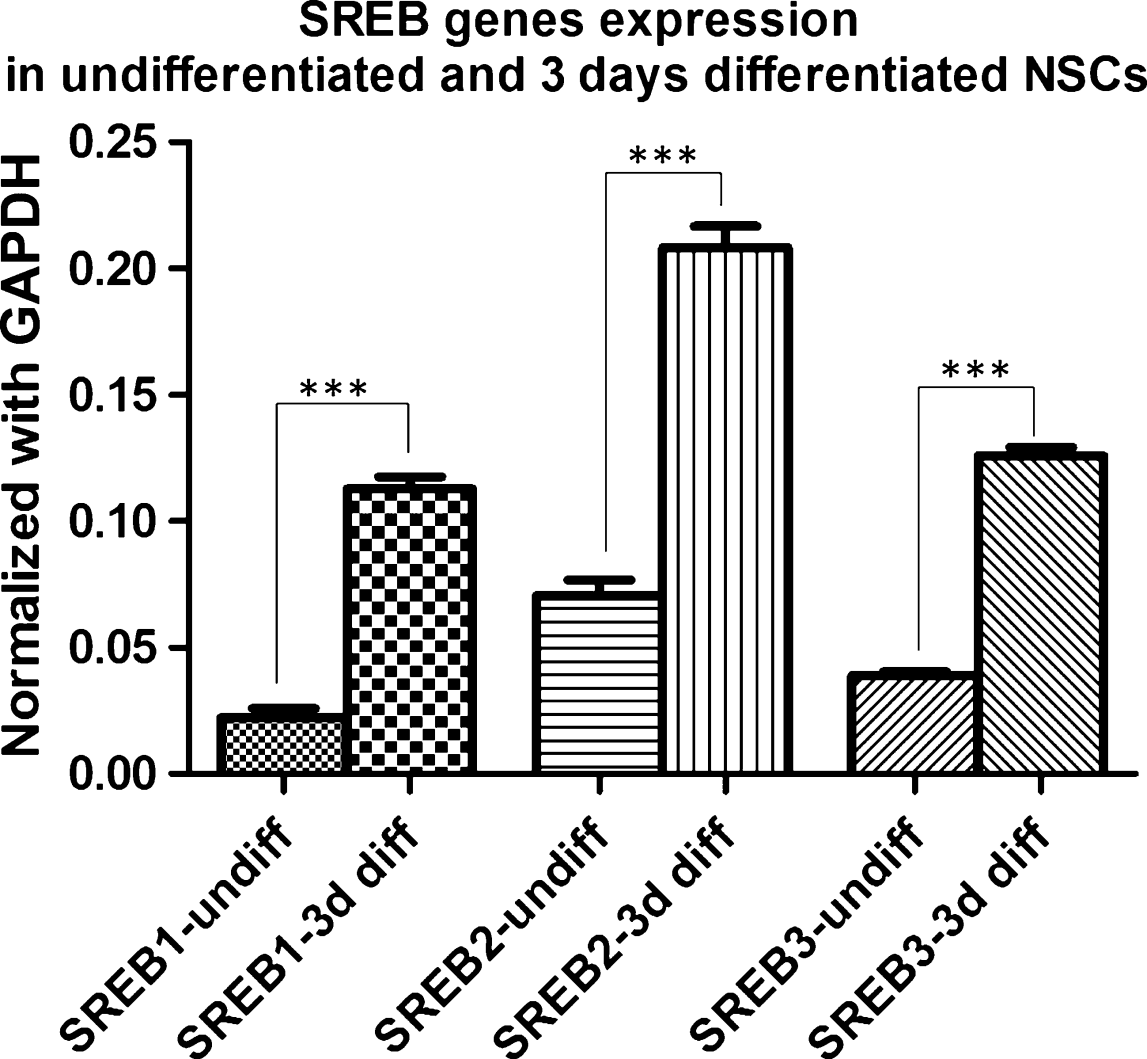
SREB2 gene expression in cultured hippocampal NSCs. A limited amount of SREB1, SREB2, and SREB3 was expressed in undifferentiated cultured NSCs. The expression of all of the SREB genes was dramatically increased during differentiation (***P < 0.0001). Data are expressed as mean values ± SEM.

The SREB2 gene is highly expressed in the dentate gyrus of the adult mouse brain. Abundant evidence shows that neural stem cells exist in the subgranule zone of the adult dentate gyrus and give rise to new granule cells that integrate into hippocampal neuronal circuits. There is little information about SREB2 gene expression during NSC differentiation. Due to the limited number of NSCs in the dentate gyrus, the quantitative analysis of SREB gene expression was assayed by the use of *in vitro* cultured hippocampal NSCs. The hippocampal NSCs were isolated from post-natal day 5 mice. SREB gene expression was examined during NSC proliferation and differentiation in three independent experiments. All three SREB genes (SREB1, 2 and 3) showed very low expression during NSC proliferation. SREB gene expression was dramatically increased at 3 days after NSC differentiation (SREB1 expression: undifferentiated vs. differentiated, two-tailed *t* = 25.25, *P* < 0.0001; SREB2 expression: undifferentiated vs. differentiated, two-tailed *t* = 22.74, *P* < 0.0001; SREB3 expression: undifferentiated vs. differentiated, two-tailed *t* = 42.07, *P* < 0.0001; Fig. 2). SREB2 had the highest expression compared with the other SREB family members. These data suggest a specific function of SREB2 in the NSC differentiation process.

### Over-expression of SREB2 inhibits hippocampal adult neurogenesis

**Fig. 3:**
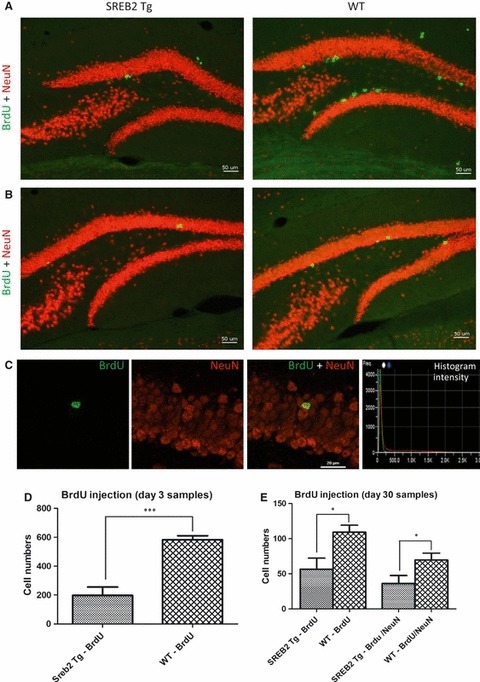
Severe deficits of hippocampal adult neurogenesis in SREB2 Tg mice. (A) Representative images of BrdU immunostaining in the dentate gyrus at 3 days after BrdU injection. Newly proliferated cells in the subgranule zone of the dentate gyrus were labeled by BrdU (green). (B) Representative images of BrdU and NeuN double staining in the dentate gyrus at 30 days after BrdU injection. Surviving cells were BrdU-positive (green). BrdU and NeuN double-stained cells (green and red) represented newly generated mature neurons. (C) Confocal microscopy analysis of double-stained BrdU/NeuN cells in SREB2 Tg and WT mouse samples. (D) Quantitative analysis of BrdU-positive cells in the subgranule zone revealed a significant decrease in new cell proliferation in SREB2 Tg mice compared with WT littermates at 3 days after BrdU injection (SREB2 Tg: 196.8 ± 58.74, *n* = 5; WT: 583.2 ± 27.65, *n* = 5; ***P < 0.001). (E) Quantitative analysis of BrdU-positive cells (SREB2 Tg: 56.40 ± 15.94, *n* = 5; WT: 109.2 ± 9.932, *n* = 5; *P < 0.05) and BrdU-positive/NeuN-positive cells (SREB2 Tg: 36.00 ± 11.54, *n* = 5; WT: 69.60 ± 9.786, *n* = 5; *P < 0.05) within the dentate gyrus revealed a significant reduction of new neuron survival and differentiation in SREB2 Tg mice at 30 days after BrdU injection. Data are expressed as mean values ± SEM.

To investigate the functional role of SREB2 in adult neurogenesis, SREB2 Tg mice were employed in these studies. To visualize newly generated cells in the adult hippocampus, BrdU was administered via i.p. injection to adult SREB2 Tg mice and their WT littermates. As a thymidine analog, BrdU can cross the blood–brain barrier and incorporate into dividing cells. To assess NSC proliferation, BrdU-positive cells were counted in the subgranule zone of dentate gyrus samples collected at 3 days after injection. There was a significant decrease in BrdU-positive cells counted in the subgranule zone of SREB2 Tg mice compared with their WT littermates (SREB2 Tg: 196.8 ± 58.74, *n* = 5; WT: 583.2 ± 27.65, *n* = 5; two-tailed *t* = 5.952, *P* = 0.0003; Fig. 3A and D). Hippocampal NSCs can differentiate into either neurons or glial cells over a period of approximately 4 weeks (Abrous *et al.*, 2005). To examine how many of the newborn cells adopted a neuronal fate, brains were sampled at 30 days after the BrdU injection, and BrdU/NeuN double staining was carried out to distinguish newly generated neurons from the surviving new cells (Fig. 3B and C). BrdU immunostaining showed a significant decrease of BrdU-positive cell numbers in SREB2 Tg mice relative to WT mice (SREB2 Tg: 56.40 ± 15.94, *n* = 5; WT: 109.2 ± 9.932, *n* = 5; two-tailed *t* = 2.811, *P* = 0.0228). BrdU/NeuN double staining was confirmed by confocal analysis, which showed a significant reduction in the survival of newborn neurons in SREB2 Tg mice (SREB2 Tg: 36.00 ± 11.54, *n* = 5; WT: 69.60 ± 9.786, *n* = 5; two-tailed *t* = 2.521, *P* = 0.0357; Fig. 3E). However, comparing the number of differentiated neurons with the surviving cells, the ratio of surviving cells that adopted a neuronal fate was similar between the SREB2 Tg and WT mice, suggesting that the reduced number of new neurons in SREB2 Tg mice is the consequence of reduced stem cell proliferation. These data suggest that the profound deficits of adult neurogenesis in SREB2 Tg mice begin at the progenitor cell proliferation stage.

### Null mutation of SREB2 enhances hippocampal adult neurogenesis

**Fig. 4:**
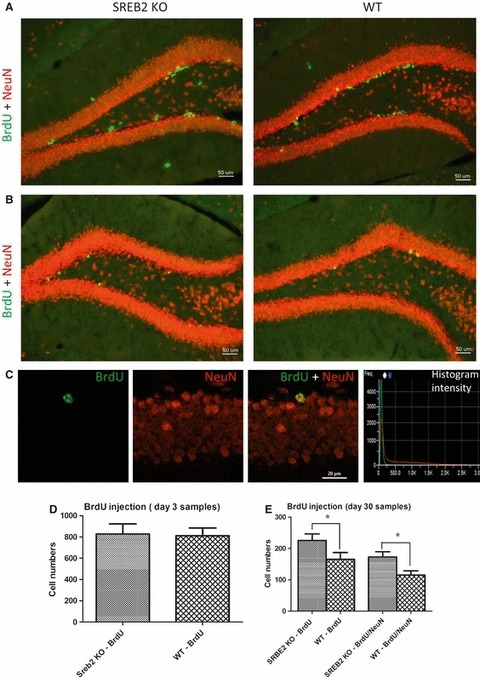
Enhanced new neuron survival in hippocampal adult neurogenesis in SREB2 KO mice. (A) Representative images of BrdU immunostaining in the dentate gyrus at 3 days after BrdU injection in SREB2 KO mice and WT control mice. BrdU-positive cells (green) in the subgranule zone of the dentate gyrus labeled the newly-proliferating cells. (B) Representative images of BrdU and NeuN double staining in the dentate gyrus at 30 days after BrdU injection in SREB2 KO mice and their littermate controls. BrdU-positive cells (green) labeled the total surviving cells, and BrdU and NeuN double-stained cells (green and red) labeled the newly-generated mature neurons. (C) Confocal microscopy analysis of double-stained BrdU/NeuN cells in SREB2 KO/WT mice. (D) Quantitative analysis of BrdU-positive cells from the subgranule zone revealed no differences in new cell proliferation in SREB2 KO mice compared with WT littermates at 3 days after BrdU injection (SREB2 KO: 829.0 ± 93.36, *n* = 6; WT: 812.0 ± 72.86, *n* = 6; *P* = 0.8887). (E) Quantitative analysis of BrdU-positive cells (SREB2 KO: 229.2 ± 20.81, *n* = 5; WT: 161.6 ± 19.66, *n* = 5; *P < 0.05) and BrdU-positive/NeuN-positive cells (SREB2 KO: 172.8 ± 16.79, *n* = 5; WT: 115.2 ± 13.66, *n* = 5; *P < 0.05) in the dentate gyrus revealed significant increases in new neuron survival and differentiation during adult neurogenesis in SREB2 KO mice at 30 days after BrdU injection. Data are expressed as mean values ± SEM.

To investigate the impact of the loss of SREB2 gene function, adult neurogenesis was also assessed in SREB2 KO mice by using BrdU labeling. In SREB2 KO mice, compared with WT littermates, there was no significant difference in BrdU-positive cell numbers in the dentate gyrus subgranule zone at 3 days after BrdU administration (SREB2 KO: 829.0 ± 93.36, *n* = 6; WT: 812.0 ± 72.86, *n* = 6; two-tailed *t* = 0.1435, *P* = 0.8887; Fig. 4A and D). However, at 30 days after BrdU injection, we found a significant increase of BrdU-positive cells in the dentate gyrus of SREB2 KO mice compared with WT littermates (SREB2 KO: 229.2 ± 20.81, *n* = 5; WT: 161.6 ± 19.66, *n* = 5; two-tailed *t* = 2.361, *P* = 0.045). Double immunostaining with the neuronal marker NeuN demonstrated that most of the BrdU-positive cells had neuronal features and the number of surviving new neurons was significantly increased in SREB2 KO mice compared with WT littermates (SREB2 KO: 172.8 ± 16.79, *n* = 5; WT: 115.2 ± 13.66, *n* = 5; two-tailed *t* = 2.661, *P* = 0.0288; Fig. 4B, C and E). The data indicate enhanced hippocampal adult neurogenesis in SREB2 KO mice, especially in the stage of new neuron survival/differentiation.

### Morphologic deficits of newly generated neurons in SREB2 Tg mice dentate gyrus region

**Fig. 5:**
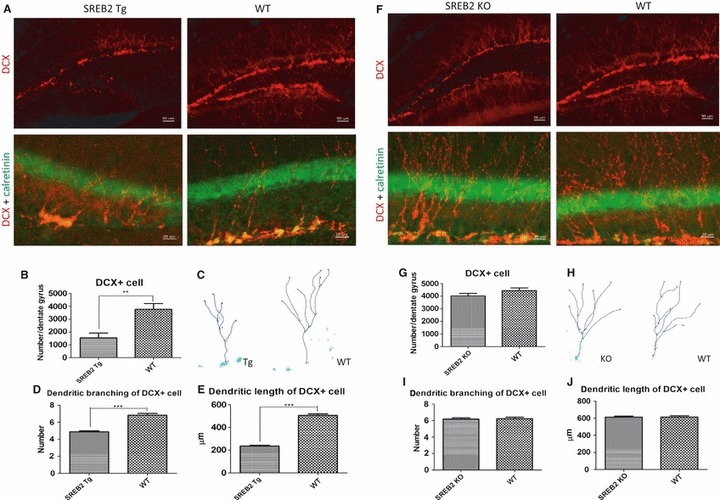
Dendrite morphology deficits in newly-generated neurons in SREB2 Tg mice. (A) Representative images of DCX immunostaining in the dentate gyrus of SREB2 Tg mice and WT littermates. DCX and calretinin double immunostaining showed the co-localization of the two immature neuron markers in differentiated new neurons. (B) A significant reduction of DCX-positive cells was found in the SREB2 Tg mouse dentate gyrus compared with WT mice (SREB2 Tg: 1539 ± 380.0, *n* = 4; WT: 3771 ± 450.7, *n* = 4; **P < 0.01). (C) Representative dendritic morphology of DCX-positive cells in SREB2 Tg and WT mice. (D) and (E) Quantitative morphology analysis of DCX-positive cells revealed significant reductions of dendritic branching (SREB2 Tg: 4.8 ± 0.1, *n* = 40; WT: 6.8 ± 0.2, *n* = 40; eight neurons were analyzed from each mouse, five mice were in each group; ***P < 0.001) and total dendritic length (SREB2 Tg: 235.5 ± 7.1 μm, *n* = 40; WT: 506.3 ± 13.1 μm, *n* = 40, ***P < 0.0001) in SREB2 Tg mice compared with WT control mice. (F) Representative images of DCX immunostaining in the dentate gyrus of SREB2 KO mice and WT littermate controls. Co-localization of DCX and calretinin expression is visualized in new differentiated neurons in the dentate gyrus. (G) No differences were observed in the number of DCX-positive cells in SREB2 KO mice compared with WT littermates (SREB2 KO: 4005 ± 213.2, *n* = 4; WT: 4437 ± 221.1, *n* = 4; *P* = 0.2). (H) Representative dendritic morphology of DCX-positive cells in SREB2 KO and WT mice. (I) and (J) No significant differences in dendrite morphology of either dendritic branching (SREB2 KO: 6.2 ± 0.2, *n* = 40; WT: 6.2 ± 0.2, *n* = 35; eight or seven neurons were analyzed from each brain, five mice of each genotype were assayed; *P* = 0.8234) or total dendritic length (SREB2 KO: 612.9 ± 11.5 μm, *n* = 40; WT: 614.2 ± 15.0 μm, *n* = 35; *P* = 0.9449) were observed in DCX-positive cells in SREB2 KO mice compared with their littermate WT controls. Data are expressed as mean values ± SEM.

Doublecortin is a reliable and specific marker with expression that begins at the neuroblast stage in the neuronal lineage and lasts during neuronal maturation. DCX expression is retained only within areas of active neurogenesis and it is rarely expressed outside these regions (Nacher *et al.*, 2001; Brown *et al.*, 2003; Couillard-Despres *et al.*, 2005). DCX immunohistochemical labeling indicated a significant reduction of DCX-positive cells in the dentate gyrus of SREB2 Tg mice (SREB2 Tg: 1539 ± 380.0, *n* = 4; WT: 3771 ± 450.7, *n* = 4; two-tailed *t* = 3.786, *P* = 0.0091; Fig. 5A and B), whereas no obvious difference in DCX-positive cell numbers was observed in SREB2 KO mice compared with their WT littermates (SREB2 KO: 4005 ± 213.2, *n* = 4; WT: 4437 ± 221.1, *n* = 4; two-tailed *t* = 1.407, *P* = 0.2; Fig. 5F and G). These results were consistent with the BrdU incorporation experiment data. DCX is a microtubule-associated protein that is distributed throughout the dendritic cytoplasm and is considered a dendritic morphology marker in newborn neurons. To distinguish DCX-labeled post-mitotic new neurons from precursor daughter cells of NSCs in SREB2 mutant mice, we double-stained DCX with calretinin – another immature neuronal marker that is only transiently expressed at the post-mitotic stage of newborn neurons (Brandt *et al.*, 2003). DCX and calretinin double staining showed the co-localization of these two markers in differentiated new neurons that displayed complex dendritic morphology extending into the molecular layer (Fig. 5A and F). The quantitative morphologic analysis of differentiated DCX-positive cells revealed that the newly generated neurons in SREB2 Tg mice had less dendrite branching (SREB2 Tg: 4.8 ± 0.1, *n* = 40; WT: 6.8 ± 0.2, *n* = 40; two-tailed *t* = 8.112, *P* < 0.0001) and shorter dendrite length (SREB2 Tg: 235.5 ± 7.1 μm, *n* = 40; WT: 506.3 ± 13.1 μm, *n* = 40; two-tailed *t* =18.23, *P* < 0.0001) compared with WT littermates (Fig. 5C–E). In SREB2 KO mice, newly generated neurons showed normal dendrite branching (SREB2 KO: 6.2 ± 0.2, *n* = 40; WT: 6.2 ± 0.2, *n* = 35; two-tailed *t* = 0.06939, *P* = 0.8234) and neurite extension (SREB2 KO: 612.9 ± 11.5 μm, *n* = 40; WT: 614.2 ± 15.0 μm, *n* = 35; two-tailed *t* = 0.06939, *P* = 0.9449; Fig. 5H–J). These results indicate that over-expression of SREB2 disrupts normal neuron differentiation in adult neurogenesis, and the newly generated neurons in SREB2 Tg mice may not exhibit mature neuronal function due to reduced dendritic complexity.

### Dentate gyrus-enriched gene expression is down-regulated in SREB2 Tg mice hippocampus

**Fig. 6:**
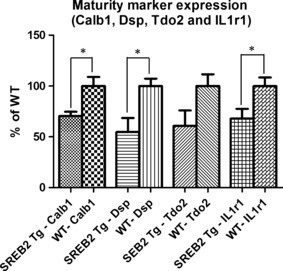
Dentate gyrus granule cell maturity gene expression profiling in SREB2 Tg mice. Quantitative reverse transcription-PCR data revealed a significant decrease of calbindin (Calb1), desmoplakin (Dsp), and interleukin-1 receptor type 1 (IL1r1) expression in the SREB2 Tg mouse (*n* = 6) hippocampus compared with WT controls (*n* = 6) (*P < 0.05). Tryptophan-2,3-dioxygenase (Tdo2) expression also showed a trend toward down-regulation in SREB2 Tg mice. Data are expressed as mean values ± SEM.

As a distinct subregion of the hippocampaus, the dentate gyrus has a unique morphology, connectivity, electrophysiological properties and molecular atlas. DNA microarrays have identified calbindin, desmoplakin, tryptophan-2,3-dioxygenase and interleukin-1 receptor type 1 as dentate gyrus enriched genes (Lein *et al.*, 2004; Hagihara *et al.*, 2009). The altered expression of these genes is associated with a disturbance of dentate gyrus function (Yamasaki *et al.*, 2008; Kobayashi *et al.*, 2010). Quantitative reverse transcription-PCR methods were employed to analyze the gene expression in SREB2 Tg mouse hippocampal samples. There was a significant decrease of calbindin, desmoplakin, and interleukin-1 receptor type 1 expression in SREB2 Tg mouse samples compared with WT littermates (calbindin expression: SREB2 Tg vs. WT, *n* = 6 of each group, two-tailed *t* = 2.981, *P* = 0.0138; desmoplakin expression: SREB2 Tg vs. WT, *n* = 6 of each group, two-tailed *t* = 2.884, *P* = 0.0163; interleukin-1 receptor type 1 expression: SREB2 Tg vs. WT, *n* = 6 of each group, two-tailed *t* = 2.511, *P* = 0.0309). Tryptophan-2,3-dioxygenase expression also showed a trend of down-regulation in SREB2 Tg mice (SREB2 Tg vs. WT, *n* = 6 of each group, two-tailed *t* = 2.060, *P* = 0.0663; Fig. 6). As these genes have been characterized as calcium-binding proteins, structural proteins, receptors and metabolic enzymes, down-regulation of these genes in SREB2 Tg mice may indicate that the normal microenvironment normally supporting adult neurogenesis in the dentate gyrus is disturbed.

### Reciprocal behavioral phenotypes of cognition in SREB2 Tg and KO mice

**Fig. 7:**
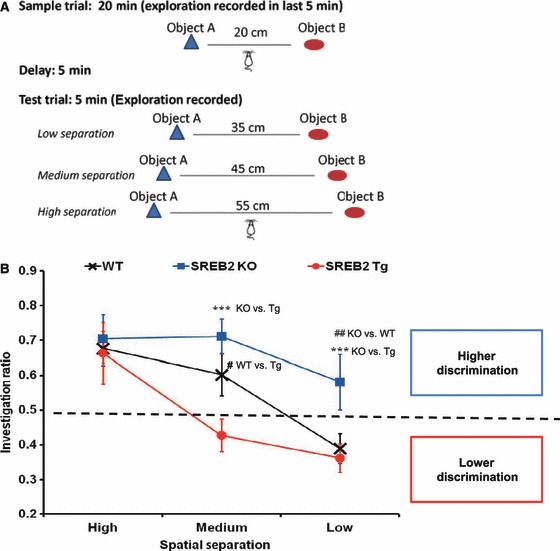
Decreasing or increasing SREB2 gene expression causes respective reciprocal changes in a spatial pattern separation test. (A) Schematic representation of the spatial pattern separation test. (B) SREB2 Tg and SREB2 KO mice showed reciprocal phenotypes in the discrimination of distance changes in the spatial pattern separation test (two-way repeated-measures anova, Genotype × Separation, *F*_4,54_ = 3.16, *P* = 0.02). All groups of the mice (WT, *n* = 8; SREB2 KO, *n* = 8; SREB2 Tg, *n* = 8) could discriminate a high spatial separation between the stimulus objects. Performance was significantly impaired in the SREB2 Tg mice at a medium separation compared with SREB2 KO and WT mice (*post-hoc* WT vs. Tg, ^#^*P* < 0.05; KO vs. Tg, ***P < 0.001). Both SREB2 Tg and WT mice could not discriminate any difference at a low separation compared with the SREB2 KO mice (*post-hoc* KO vs. WT, ^##^*P* < 0.01; KO vs. Tg, ***P < 0.001. Data are expressed as mean values ± SEM.

The SREB2 Tg and SREB2 KO mice, and their WT littermates were examined simultaneously in behavioral tests. The same cohort of mice was used in all of the behavior assays. There was a 2 week interval between the pattern separation test and the delayed Y-maze test. As there were no significant differences in any measures between WT littermates of SREB2 Tg mice and SREB2 KO mice, we combined WT data from mixed WT littermates of SREB2 Tg and KO mice. Dentate gyrus-dependent spatial pattern separation was measured with a variation of a metric spatial-processing task (Hunsaker *et al.*, 2009). All mice displayed a habituation to the stimulus objects during the sample phase of the trial as demonstrated by a decrease in investigation duration during the 20 min interval. During the test phase of the trial, an increase in investigation duration (compared with the last 5 min of the sample phase) was interpreted as an ability of the mouse to detect the novel spatial orientation of the objects. As such, investigation ratios of more than 0.5 indicate the ability of an animal to discriminate a change in spatial orientation (Fig. 7A). As a control, all of the mice were tested in a static spatial separation trial during which there was no change in the object location between the sample and test trials. During this control, all of the mice had shorter investigation durations during the test (investigation ratios: WT, 0.39 ± 0.04, *n* = 8; SREB2 KO, 0.39 ± 0.08, *n* = 8; SREB2 Tg, 0.36 ± 0.04, *n* = 8). Under the test conditions of spatial separation, a repeated-measures anova with Genotype as a between-group factor and Separation as within-group factor yielded a significant interaction (Genotype × Separation, *F*_4,54_ = 3.16, *P* = 0.02). At a high spatial separation (35 cm difference), all three genotypes of mice were able to discriminate the difference in spatial orientation of the objects between the sample and test trials and showed longer investigation durations (investigation ratios: WT, 0.68 ± 0.05, *n* = 8; SREB2 KO, 0.70 ± 0.07, *n* = 8; SREB2 Tg, 0.66 ± 0.09, *n* = 8, Fig. 7B). At a medium spatial separation (25 cm difference), the WT and SREB2 KO mice were able to discriminate the difference in spatial orientation of the objects but the performance of the SREB2 Tg mice was significantly impaired (investigation ratios: WT, 0.60 ± 0.06, *n* = 8; SREB2 KO, 0.71 ± 0.05, *n* = 8; SREB2 Tg, 0.43 ± 0.05, *n* = 8; *post-hoc* WT vs. Tg, *P* < 0.05; KO vs. Tg, *P* < 0.001, Fig. 7B). When the spatial separation was further decreased to a short distance (15 cm difference), both WT and SREB2 Tg mice showed a lack of spatial pattern separation ability that was significantly impaired compared with the SREB2 KO mice, which retained good discrimination (investigation ratios: WT, 0.39 ± 0.04, *n* = 8; SREB2 KO, 0.58 ± 0.08, *n* = 8; SREB2 Tg, 0.36 ± 0.04, *n* = 8; *post-hoc* KO vs. WT, *P* < 0.01; KO vs. Tg, *P* < 0.001, Fig. 7B). The SREB2 KO mice showed enhanced spatial pattern separation capacity under increasingly difficult discrimination conditions, which is consistent with their enhanced neurogenesis phenotype.

**Fig. 8:**
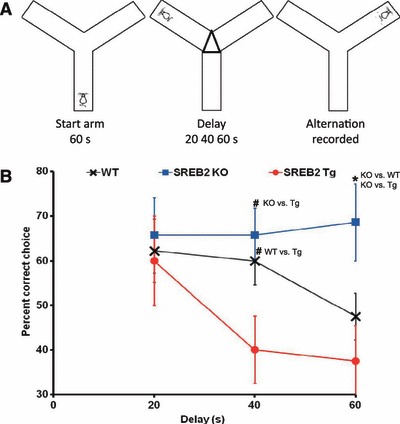
SREB2 Tg and SREB2 KO mice exhibit reciprocal phenotypes in delayed spontaneous alternation in Y-maze. (A) Schematic representation of the spontaneous delayed alternation in Y-maze task used to evaluate spatial working memory. (B) The relative SREB2 expression in SREB2 Tg and SREB2 KO mice resulted in respective significant effects in their working memory performance in the delayed Y-maze test (anova, genotype: *F*_2,23_ = 5.59, *P* = 0.01). Working memory performance was similar for all three genotypes (WT, *n* = 8; SREB2 KO, *n* = 8; SREB2 Tg, *n* = 8) at a short delay of 20 s. When the delay was increased to 40 s, the SREB2 Tg mice were impaired compared with WT and SREB2 KO mice (*post-hoc* KO vs. Tg, WT vs. Tg, ^#^*P* < 0.05). At a long delay of 60 s, the SREB2 KO mice performed significantly better than the other two groups (*post-hoc* KO vs. WT, KO vs. Tg, *P < 0.05). Data are expressed as mean values ± SEM.

Spatial working memory was examined using the Y-maze delayed spontaneous alternation task at delay intervals of 20, 40, or 60 s (Fig. 8A). An anova analysis yielded a significant effect of Genotype in the Y-maze test (*F*_2,23_ = 5.59, *P* = 0.01). At a 20 s delay, WT, SREB2 Tg and SREB2 KO mice all performed at better than random alteration (33.3%) (% correct: WT, 62.2 ± 7.0, *n* = 8; SREB2 KO, 65.7 ± 8.4, *n* = 8; SREB2 Tg, 60 ± 10, *n* = 8, Fig. 8B). At a 40 s delay, SREB2 Tg mice showed a significant impairment compared with the other two groups of mice (% correct: WT, 60 ± 5.3, *n* = 8; SREB2 KO, 65.7 ± 7.2, *n* = 8; SREB2 Tg, 40 ± 7.6, *n* = 8; *post-hoc* WT vs. Tg, *P* < 0.05; KO vs. Tg, *P* < 0.05, Fig. 8B). At the longest delay tested (60 s), both SREB2 Tg and WT mice showed impaired performance compared with the SREB2 KO mice, which still performed at above random alternation (% correct: WT, 47.5 ± 5.3, *n* = 8; SREB2 KO, 68.6 ± 8.6, *n* = 8; SREB2 Tg, 37.5 ± 8, *n* = 8; *post-hoc* KO vs. WT, *P* < 0.05; KO vs. Tg, *P* < 0.05, Fig. 8B). Taken together, SREB2 Tg mice have working memory impairments at both moderate and long delays, whereas SREB2 KO mice have enhanced working memory performance in this task.

## Discussion

SREB2 is an orphan G-protein-coupled receptor identified as a risk factor for schizophrenia. Although SREB2 is a potential novel drug target for psychiatric disorders, its functional role in normal brain and in pathological conditions remains elusive. Accumulated evidence has shown that hippocampal dysfunction is one of the core pathophysiological features of schizophrenia (Weinberger, 1999; Harrison, 2004; Boyer *et al.*, 2007; Tamminga *et al.*, 2010). Not surprisingly, the neonatal ventral hippocampus lesion model of schizophrenia has profound behavioral deficits similar to those clinically observed in human patients (Tseng *et al.*, 2009). In this study, we report for the first time a functional role of SREB2 in the hippocampal formation in regulating adult neurogenesis and neurogenesis-related cognitive functions.

Adult neurogenesis incorporates the stages of neural progenitor proliferation, neural differentiation/maturation and, finally, integration of the adult-born neurons into the surrounding network. SREB2 Tg mice displayed a robust reduction in progenitor cell proliferation and a consequential decrease in newborn neuron number, whereas the SREB2 KO mice showed an increase in the survival of newborn neurons with no change in the initial rate of their proliferation. Unlike programmed and orchestrated events in embryonic neuronal development, adult neurogenesis is an individual cellular process that requires the maintenance of a permissive microenvironment (Li & Pleasure, 2010). As such, adult neurogenesis can be modified by many intrinsic and extrinsic factors and programs (Suh *et al.*, 2009; Ming & Song, 2011). In the adult brain, SREB2 expression is only detected in neurons, whereas during embryonic development SREB2 is abundantly expressed during the early phase of neuronal differentiation, which points to the developmental function of this gene in differentiation processes (Hellebrand *et al.*, 2001; Jeon *et al.*, 2002). These observations were recapitulated by our *in vitro* experiments using hippocampus-derived stem cells that showed very low levels of SREB2 mRNA expression in the progenitor cell proliferation stage, whereas expression quickly increased during the neuronal differentiation stage. The increased survival of newborn neurons (with no change in cell proliferation) in SREB2 KO mice indicates the potentially strong, intrinsic, inhibitory effect of SREB2 in new neuron differentiation and survival. However, in our SREB2 Tg mice, the over-expressed SREB2 gene was driven by a calcium/calmodulin dependent protein kinaseII alpha (CaMKIIα) promoter that restricted the transgene expression to post-natal neurons in the forebrain region. As the SREB2 transgene expression is very limited, or even absent, in progenitor cells, one possibility is that over-expressed SREB2 in the neurons adjoining the progenitor cells may alter the extrinsic environment of a neurogenic niche toward more inhibition for progenitor cell proliferation and it may consequently lower the new neuron number in the dentate gyrus. We examined the expression of the dentate gyrus maturation marker genes calbindin, desmoplakin, tryptophan-2,3-dioxygenase, and interleukin-1 (Kobayashi *et al.*, 2010). The expression of all of these genes was significantly decreased or showed trends toward down-regulation in the SREB2 Tg mouse hippocampus sample. These data support the idea that the over-expressed SREB2 disrupted the normal microenvironment that regulates adult neurogenesis. Taken together, these results indicate that SREB2 may work as both an intrinsic and extrinsic negative regulatory factor to influence neurogenesis and, consequentially, shape hippocampal function in the adult brain.

During normal adult neurogenesis, newly generated neurons are required to functionally integrate into the pre-existing neuronal network (Ramirez-Amaya *et al.*, 2006). Appropriate neuronal migration, neurite extension, and path-finding of newborn neurons are critical for optimal neuronal circuit formation (Toni *et al.*, 2008). The dendrite morphology analysis that we conducted in SREB2 Tg mice of their differentiated DCX-positive neurons demonstrated distinct morphological deficits in these newly generated neurons. The diminished dendritic structure of the adult-born neurons has the potential to impair neuronal circuitry in the hippocampus and may ultimately contribute to the functional behavioral deficits in SREB2 Tg mice. In contrast, the adult-born neurons in the SREB2 KO mice showed normal dendritic morphology, which indicates normal functional integration of their new neurons into the neuronal network. Further experiments are needed to elucidate the specific molecular mechanisms and down-stream signaling of SREB2 that directly regulate newborn neuron survival and morphogenesis of adult-born neurons.

It is increasingly clear that, whereas the entire hippocampus creates representations of contexts and episodes, the dentate gyrus is especially involved in the ability to discriminate between fine spatial/contextual changes in the environment and to allow these representations to be recalled with fidelity (McHugh *et al.*, 2007; Goodrich-Hunsaker *et al.*, 2008; Hunsaker & Kesner, 2008; Hunsaker *et al.*, 2008). This cognitive ability to disambiguate complex, but similar, information is called spatial pattern separation. Spatial pattern separation may be the only cognitive feature known to rely exclusively on both intact dentate gyrus function and adult neurogenesis in the dentate gyrus (Deng *et al.*, 2010; Sahay *et al.*, 2011; Yassa & Stark, 2011). It has been suggested that disruption in the pattern separation function of the dentate gyrus may underlie some of the clinical features of schizophrenia (Tamminga *et al.*, 2010). Elevation of SREB2 expression in the SREB2 Tg mice leads to deficits in spatial pattern separation, as demonstrated by the lack of discrimination by the mice of the metric relationship between two objects under challenging conditions. Our static spatial separation test result indicates that these mice have intact short-term memory for recognizing the objects used in the test when the spatial relationship between them is unaltered. Therefore, the discrimination deficits that we observed in the transgenic mice are not mnemonic in nature. Consistent with their deficits in neurogenesis, the SREB2 Tg mice are impaired primarily on the medium- and low-separation trials of the task, which place a larger demand on pattern separation capacity. Conversely, SREB2 KO mice, which have enhanced survival of newborn neurons in the dentate gyrus, display superior spatial pattern separation abilities in that they can distinguish even relatively small changes in the metric relationships of objects that are undetectable by both SREB2 Tg and WT mice.

The role of adult neurogenesis in the dentate gyrus in working memory function has not been as clearly elucidated as in spatial pattern separation. Whereas some investigators have shown that a reduction of neurogenesis can impair working memory performance, others have found either no change or a paradoxical improvement in working memory (Winocur *et al.*, 2006; Saxe *et al.*, 2007; Clelland *et al.*, 2009; Lazic, 2010). However, this discrepancy may be due to many factors including the age of the animals studied, the range of working memory delays tested (or lack thereof) and the spatial parameters of the tasks used in the different laboratories. Here, we used a standard Y-maze to test working memory with the critical variable being the temporal delay between the sample and choice phases of a delayed spontaneous alternation task. Under these conditions, SREB2 Tg mice had normal rates of alternation when the delays were relatively low, but did not perform above random when the delay was increased to a moderate interval at which the WT mice still performed well. Conversely, SREB2 KO mice demonstrated enhanced performance in this task at delays at which the WT mice showed random alternation. Based on our results, it appears that the level of hippocampal adult neurogenesis impacts both spatial and temporal aspects of cognition.

Accumulating evidence is establishing the role of adult neurogenesis in the pathophysiology of psychiatric disorders (DeCarolis & Eisch, 2010; Pickard, 2011). A recent report showed that NSC proliferation is decreased in the brains of patients with schizophrenia (Reif *et al.*, 2006). Three widely reported schizophrenia susceptibility genes, disrupted in schizophrenia 1 (DISC1), neuregulin 1, and dysbindin, have been shown to regulate new neuron development in adult brain (Falls, 2003; Duan *et al.*, 2007; Kim *et al.*, 2009; Nihonmatsu-Kikuchi *et al.*, 2011). In addition, abnormalities in a number of signaling pathways involved in the regulation of embryonic neural development have been reported in schizophrenia that implicate the ongoing role of these molecules in adult neurogenesis (Toro & Deakin, 2007). Disruptions in the neurogenesis-dependent pattern separation function of the dentate gyrus have been suggested to underlie some of the clinical features of schizophrenia (Tamminga *et al.*, 2010). The fact that SREB2 is a schizophrenia susceptibility gene indicates that the function of SREB2 in adult neurogenesis may contribute to the etiology of psychiatric diseases. A further characterization of the signal pathway of SREB2 focusing on adult neurogenesis may lead to advanced understanding of the etiological/pathophysiological role of SREB2 in these diseases. Targeting SREB2 signaling may provide a powerful and novel approach to ameliorate the cognitive symptoms of patients with schizophrenia and other psychiatric diseases.

## Abbreviations

BrdU: bromodeoxyuridine
DCX: doublecortin
KO: knockout
NeuN: neuronal specific nuclear protein
NSC: neural stem cell
PBS: phosphate-buffered saline
PCR: polymerase chain reaction
SREB: super-conserved receptor expressed in brain
Tg: transgenic
WT: wild-type
